# DNA-PKcs promotes alcohol-related liver disease by activating Drp1-related mitochondrial fission and repressing FUNDC1-required mitophagy

**DOI:** 10.1038/s41392-019-0094-1

**Published:** 2019-12-06

**Authors:** Hao Zhou, Pingjun Zhu, Jin Wang, Sam Toan, Jun Ren

**Affiliations:** 1 Chinese PLA General Hospital, Medical School of Chinese PLA, 100853 Beijing, China; 2 Center for Cardiovascular Research and Alternative Medicine, University of Wyoming College of Health Sciences, Laramie, WY 82071 USA; 3 Department of Chemical Engineering, University of Minnesota-Duluth, Duluth, MN 55812 USA

**Keywords:** Gastrointestinal diseases, Cell biology

## Abstract

DNA-dependent protein kinase catalytic subunit (DNA-PKcs) is a novel housekeeper of hepatic mitochondrial homeostasis outside the DNA repair process. In this study, DNA-PKcs was upregulated in the livers of mice that were exposed to alcohol; the expression of DNA-PKcs positively correlated with hepatic steatosis, fibrosis, apoptosis, and mitochondrial damage. Functional studies revealed that liver-specific DNA-PKcs knockout (DNA-PKcs^*LKO*^) mice were protected from chronic ethanol-induced liver injury and mitochondrial damage. Mechanistic investigations established that DNA-PKcs promoted p53 activation, which elevated dynamin-related protein 1 (Drp1)-related mitochondrial fission but repressed FUN14 domain containing 1 (FUNDC1)-required mitophagy. Excessive fission and defective mitophagy triggered mtDNA damage, mitochondrial respiratory inhibition, mROS overproduction, cardiolipin oxidation, redox imbalance, calcium overload, and hepatic mitochondrial apoptosis. In contrast, the deletion of DNA-PKcs rescued these phenotypic alterations, which alleviated the susceptibility of hepatocytes to alcohol-induced cytotoxicity. Additionally, we also showed that orphan nuclear receptor subfamily 4 group A member 1 (NR4A1) was the upstream signal for DNA-PKcs activation and that the genetic ablation of NR4A1 ameliorated the progression of alcohol-related liver disease (ARLD); these results were similar to those obtained in DNA-PKcs knockout mice. Collectively, our results identified the NR4A1/DNA-PKcs/p53 axis as a novel signaling pathway responsible for ARLD pathogenesis that acts by activating Drp1-related mitochondrial fission and restricting FUNDC1-required mitophagy. The findings have potential implications for new approaches for ARLD therapy.

## Introduction

The hallmarks of alcohol-related liver disease (ARLD) include alterations in the architecture and function of mitochondria.^1^ Over the past few decades, careful research from many labs has indicated that cell death following mitochondrial dysfunction contributes to the pathogenesis of ARLD.^2^ Recent studies have found that dynamic mitochondrial processes, especially mitochondrial fission and mitophagy, are vital for liver mitochondrial homeostasis under diabetic,^3^ non-ARLD,^4^ and ischemia-reperfusion damage conditions.^5^ However, the roles of mitochondrial fission and mitophagy in alcohol-induced mitochondrial dysfunction and liver injury have rarely been studied. Findings from our group have revealed that excessive fission and defective mitophagy promote the progression of fatty liver diseases.^6,7^ Whether these molecular signals are involved in the etiology of ARLD and, if so, the regulatory mechanisms that link fission and mitophagy to ARLD remain largely elusive.

Mitochondrial fission is primarily mediated by dynamin-related protein 1 (Drp1), which resides in the cytosol and translocates to the outer mitochondrial membrane during fission.^8^ The upstream transcription activator of Drp1 is p53, which has been found to bind to the Drp1 promoter and promote Drp1 transcription.^9^ Considering the established deleterious role of p53 in ARLD,^10^ we explored whether p53 is implicated in Drp1-related fission in ARLD. Three receptors have been found to be related to mitophagy: FUN14 domain containing 1 (FUNDC1), BCL2 interacting protein 3 (Bnip3), and Parkin.^11,12^ Many studies have described the critical functions of Bnip3 and Parkin in liver protection under diabetic and non-ARLD conditions.^13,14^ Additionally, according to our previous studies, different receptors that mediate mitophagy may influence cell fate.^15–17^ Therefore, confirming the exact role of FUNDC1-related mitophagy in ARLD is necessary. FUNDC1 is activated via posttranscriptional modification at Ser13.^18,19^ The LC3-interacting region (LIR) motif of FUNDC1 is phosphorylated by casein kinase 2 (CK2) or receptor-interacting protein 3 (Ripk3),^19,20^ and this inhibits its interaction with LC3 and prevents mitophagy activation. Given the evidence linking p53 to CK2 activation,^21^ we aimed to determine whether p53 can inactivate FUNDC1 mitophagy through CK2.

Under normal conditions, cells employ mouse double minute 2 homolog (MDM2) to degrade p53 to block p53-induced cellular apoptosis.^22^ However, the phosphorylation of p53 at Ser15 increases resistance to MDM2-mediated protein degradation. The vital initial signal for p53 phosphorylation is DNA-dependent protein kinase catalytic subunit (DNA-PKcs).^23^ DNA-PKcs and Ku70/Ku80 constitute DNA-dependent protein kinase (DNA-PK), which is a central player in DNA double-strand break (DSB) repair and genomic stability.^24^ There are two entirely different consequences of DNA-PKcs activation. In response to DNA DSB, DNA-PKcs is activated and interacts with Ku80 to mediate DBS repair.^25^ However, in instances of prolonged insult, especially in chronic metabolic damage, DNA-PKcs is activated and phosphorylates p53,^26^ which promotes mitochondrial injury through the induction of mitochondrial apoptosis in liver cancer.^27^ Accordingly, the DNA-PKcs/p53 pathway is a pro-apoptosis signal. Finally, in response to feeding and insulin signaling, DNA-PKcs has been shown to transcriptionally upregulate genes involved in lipogenesis.^28^ In addition, DNA-PKcs mediates free-fatty-acid-induced lipid accumulation in hepatocytes, which may contribute to the pathogenesis of nonalcoholic steatohepatitis.^29^ In the present study, through a DNA-PKcs loss-of-function assay, we investigated the comprehensive role of DNA-PKcs in the etiology of ARLD with a particular focus on p53 activation, Drp1-required fission, and FUNDC1-related mitophagy.

## Results

### DNA-PKcs is activated by alcohol stimulation and contributes to the pathogenesis of ARLD

**Fig. 1:**
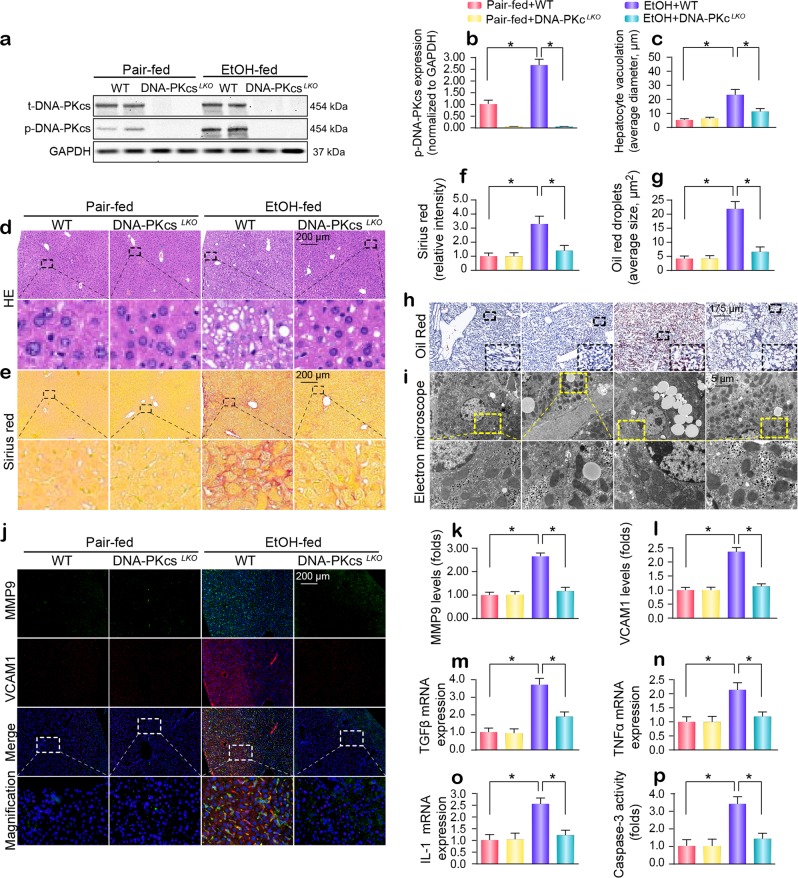
A DNA-PKcs-dependent pathway is required for the development of chronic ethanol-induced liver disease. WT and DNA-PKcs^*LKO*^ mice were allowed free access to an ethanol or control diet (*n* = 6). **a**, **b** Liver tissues from WT and DNA-PKcs^*LKO*^ mice treated with or without alcohol were analyzed using western blotting. **c**, **d** Paraffin-embedded liver sections were stained with hematoxylin and eosin. **e**, **f** Hepatic fibrosis was detected by Sirius Red staining. **g**, **h** Frozen liver sections were subjected to Oil Red O staining. **i** Ultrastructural changes in hepatocytes, especially mitochondria, in response to alcohol treatment. **j**–**l** Immunofluorescence analysis of MMP9 and VCAM1. **m**–**o** qPCR was used to explore the changes in TGFβ, TNFα and IL-1 transcription. **p** Caspase-3 activity was used to detect hepatic apoptosis. The experiments were repeated three times with similar results. The data represent the mean ± standard error of the mean. *p < 0.05.

After 16 weeks of alcohol intake, phosphorylated DNA-PKcs (p-DNA-PKcs^S2056^, an active form of DNA-PKcs) was dramatically upregulated (Fig. 1a, b). In vitro, isolation and cultivation seemed to have little influence on DNA-PKcs transcription and expression in primary hepatocytes from wild-type (WT) mice (Supplementary Fig. S1a–c), whereas alcohol treatment significantly promoted DNA-PKcs phosphorylation (Supplementary Fig. S1d, e). To determine whether active DNA-PKcs is implicated in the progression of ARLD, liver-specific DNA-PKcs knockout (DNA-PKcs^*LKO*^) mice treated with or without alcohol were used. As shown in Supplementary Table S1, chronic alcohol intake significantly increased the organ (liver and heart) weight and size (normalized to body weight) without affecting body or kidney weights. These changes were reversed by DNA-PKcs deletion. Additionally, chronic alcohol intake elicited a significant elevation in serum ALT and AST levels and the ratio of AST to ALT, and these effects of which were abolished by DNA-PKcs deletion (Supplementary Table S1).

Structurally, no significant change in liver tissue was observed between WT mice and DNA-PKcs^*LKO*^ mice under normal conditions (Fig. 1c, d). Interestingly, after chronic alcohol intake, WT mice developed severe ARLD, as witnessed by hepatocyte vacuolation (Fig. 1c, d), fibrosis (Fig. 1e, f) and steatosis (Fig. 1g, h). Ultrastructurally, in alcohol-treated mice, more lipid droplets were observed, and mitochondria were short/round in the hepatocyte cytoplasm (Fig. 1i). Strikingly, these defects were completely absent in DNA-PKcs^*LKO*^ mice.

By immunofluorescence, we found that the levels of MMP-9 and VCAM1 were upregulated in the alcohol-treated mice when compared to the WT mice (Fig. 1j–l). Similarly, the transcription of TGFβ, TNFα and IL1 was also increased in the livers of alcohol-treated mice (Fig. 1m–o). However, the loss of DNA-PKcs recused these phenotypic changes. In primary hepatocytes, under alcohol insult, alcohol also increased the transcription of inflammatory/fibrosis markers such as monocyte chemotactic protein 1 (MCP1), macrophage inflammatory protein 1α (MIP1α) and interleukin 8 (IL8) in a DNA-PKcs-dependent manner (Supplementary Fig. S1f–h). In addition, hepatocyte viability was also reduced by alcohol stress, as evidenced by increased caspase-3 activity in vivo (Fig. 1p) and in vitro (Supplementary Fig. S1i). However, the loss of DNA-PKcs prevented caspase-3 activation in the presence of alcohol stress.

### DNA-PKcs deficiency abolishes alcohol-induced hepatocyte death

**Fig. 2:**
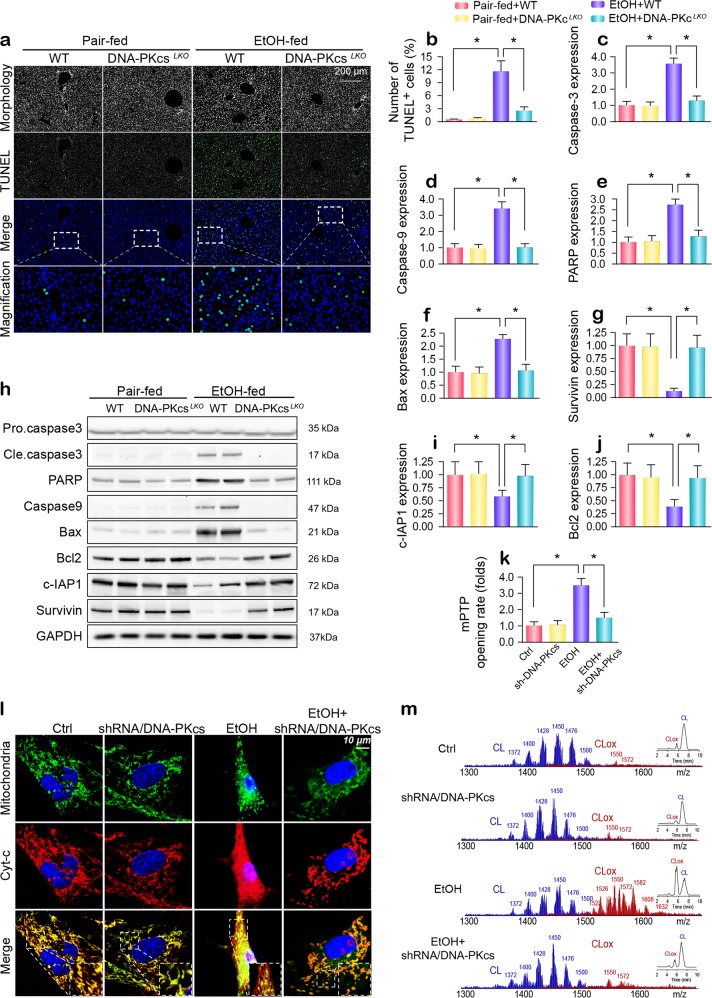
DNA-PKcs is involved in chronic ethanol-induced hepatocyte apoptosis. **a**, **b** The TUNEL assay was used to detect cellular apoptosis in vivo. **c–j** Tissue lysates from the livers of WT and DNA-PKcs^*LKO*^ of untreated or alcohol-treated mice were analyzed using western blotting to determine the expression of mitochondrial apoptosis proteins. **k** Arbitrary mPTP opening time was determined as the time when the initial TMRE fluorescence intensity decreased by half compared to the residual TMRE fluorescence intensity. **l** Immunofluorescence for cyt-c was used to detect its cellular location in hepatocytes. Mitochondria were labeled with Tom20. Nuclei were labeled by DAPI. **m** Assessment of molecular species of CL and its oxidation products. The left panel indicates nonoxidized CL species (blue) and the appearance of numerous oxidized CL species (red) after alcohol attack. Right insert: Two-dimensional chromatographic separation of nonoxidized and oxidized CL (CLox). Alcohol caused extensive CL oxidation. The experiments were repeated three times with similar results. The data represent the mean ± standard error of the mean. *p *<* 0.05.

To demonstrate the harmful effects of DNA-PKcs on liver damage, we focused on cell death, which is a primary factor responsible for the development of ARLD. Chronic alcohol stimulation augmented the TUNEL-positive ratio in the liver, and this change was rescued by the genetic ablation of DNA-PKcs (Fig. 2a, b). Next, western blot analysis demonstrated that mitochondrial apoptosis-related proteins were activated by alcohol stress and were inhibited by DNA-PKcs deletion (Fig. 2c–j). Similar changes were also observed in primary hepatocytes in vitro (Supplementary Fig. S2a–e). Cyt-c release and mPTP opening are features of mitochondrial death. In primary hepatocytes, alcohol treatment mediated mPTP opening (Fig. 2k) and promoted cyt-c leakage from the mitochondria into the cytoplasm and even into the nucleus (Fig. 2l); these effects were negated by DNA-PKcs deletion. This finding was further validated by western blotting in vivo (Supplementary Fig. S2f–h). In healthy hepatocytes, cyt-c is preferentially bound to the inner mitochondrial membrane by an association with the anionic phospholipid cardiolipin (CL),^30,31^ and cyt-c is reversibly liberated upon the peroxidation of CL.^32^ Using 2-dimensional high-performance thin-layer chromatography, we performed a global lipidomics analysis of CL (Fig. 2m), which demonstrated ~185 individual molecular species of CL in normal liver mitochondria (Supplementary Fig. 2i), of which only ~10 were oxygenated. Notably, alcohol induced the oxidation of the majority of polyunsaturated molecular species of CL, and the number of nonoxidized CL species decreased to ~94, whereas the number of oxygenated species increased to ~154 (Supplementary Fig. S2i). However, DNA-PKcs silencing reduced the level (Fig. 2m) and number (Supplementary Fig. S2i) of oxidized CL.

### Deletion of DNA-PKcs protects hepatic mitochondria against alcohol injury

**Fig. 3:**
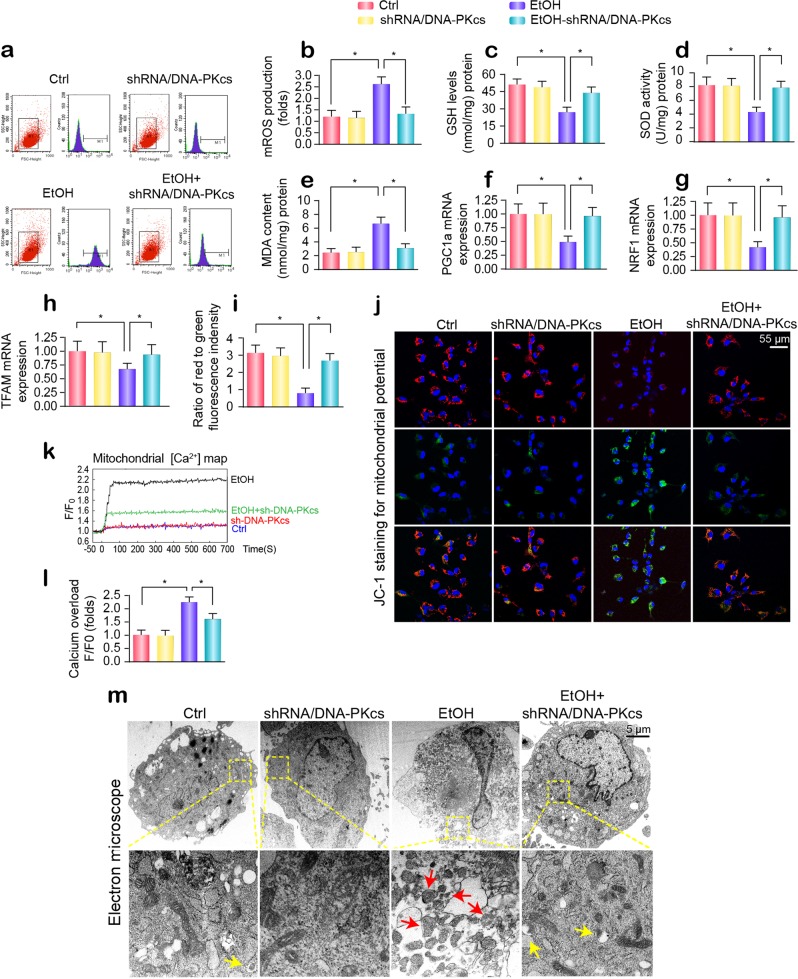
Alcohol induces liver mitochondrial damage via DNA-PKcs. **a**, **b** Mitochondrial reactive oxygen species content. The chart indicates the quantitative flow cytometry results. **c**–**e** Changes in GSH, SOD, and MDA levels, as determined by ELISA. **f**–**h** mRNA analysis of PGC-1a, NRF1, TFAM in hepatocytes transfected with shRNA/DNA-PKcs in the presence of ethanol stimulation. **i**, **j** The membrane potential was analyzed by JC1 staining. **k**, **l** The [Ca^2+^]m map, as determined by confocal microscopy using Rhod-2. The fluorescence intensity of Rhod-2 was measured at an excitation wavelength of 550 nm and an emission wavelength of 570 nm. The data (F/F0) were obtained by dividing the fluorescence intensity (F) by the fluorescence intensity at the resting level (F0) (*t* = 0) and were normalized to the control groups. **m** Representative electron microscopy (EM) images of morphological changes in mitochondria. Red arrows: fragmented or round mitochondria. Yellow arrows: mitochondria contained in lysosomes, indicative of mitophagy. The experiments were repeated three times with similar results. The data represent the mean ± standard error of the mean. *p < 0.05.

Given that CL oxidation is largely derived from mROS overproduction, we measured the changes in the mROS content. Flow cytometry showed that alcohol elevated mROS generation in primary hepatocytes (Fig. 3a, b), a result that was followed by a drop in the levels of GSH/SOD and an increase in the production of MDA (Fig. 3c–e). DNA-PKcs deficiency restored the redox balance (Fig. 3a–e). In addition to mitochondrial oxidative stress, mitochondrial biogenesis, as evidenced by PGC1a, NRF1, and TFAM transcription, was significantly inhibited by alcohol stress and was reversed to near-normal levels by DNA-PKcs deletion (Fig. 3f–h). In addition, the mitochondrial membrane potential (ΔΨm) was decreased by alcohol treatment and was maintained by DNA-PKcs silencing in hepatocytes (Fig. 3i, j). Apart from the loss of proton chemical gradients, alcohol also induced an elevation in the concentration of mitochondrial calcium ([Ca^2+^]m) (Fig. 3k, l), and this effect was inhibited by DNA-PKcs deletion. This finding indicates that alcohol-mediated mitochondrial dysfunction can be improved by DNA-PKcs deletion. Structurally, alcohol led to the formation of numerous round mitochondrial fragments of varying sizes (red arrows in Fig. 3m), as assessed by electron microscopy. However, DNA-PKcs-depleted cells retained a near-normal reticulotubular mitochondrial morphology. Of note, DNA-PKcs deficiency also caused more mitochondria to be swallowed by lysosomes, indicating that mitophagy activation was induced by DNA-PKcs deletion (yellow arrows in Fig. 3m).

### DNA-PKcs aggravates Drp1-required mitochondrial fission

**Fig. 4:**
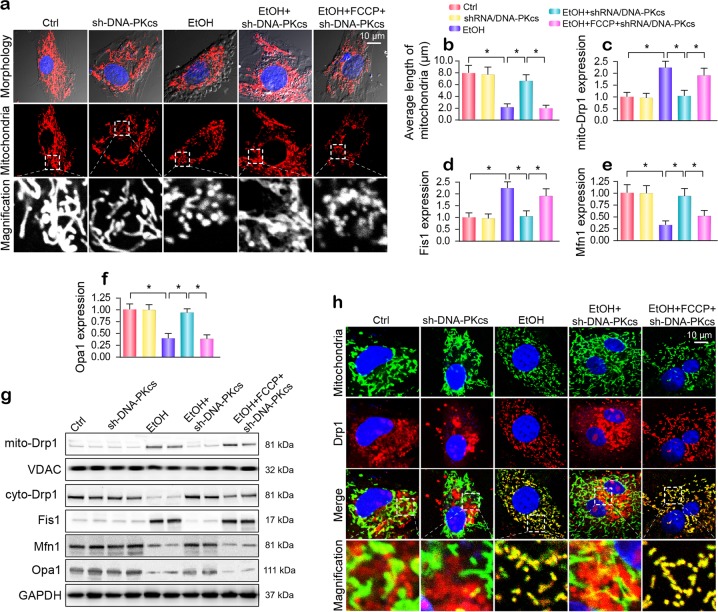
Drp1-related mitochondrial fission is modulated by DNA-PKcs and promotes mitochondrial damage under alcohol stress. **a** Immunofluorescence assay for mitochondria in hepatocytes transfected with shRNA/DNA-PKcs in the presence of ethanol stimulation. The boxed area under each micrograph is enlarged to show mitochondrial fragmentation. **b** To quantify mitochondrial fission, the average length of the mitochondria in each cell was calculated. **c**–**g** Western blotting was used to examine the alterations in mitochondrial fission-related proteins. VDAC and GAPDH were loading controls for mitochondrial proteins and cytoplasmic proteins, respectively. **h** Immunofluorescence for Drp1 in subcellular compartments after alcohol treatment. Mitochondria were labeled with Tom20. The boxed area under each micrograph is enlarged to show the overlap between Drp1 and mitochondria; fragmentation. Orange immunofluorescence indicates the binding of Drp1 to mitochondria debris. The experiments were repeated three times with similar results. The data represent the mean ± standard error of the mean. *p < 0.05.

Because more mitochondrial debris was observed under alcohol treatment, we examined whether alcohol induces mitochondrial fission. As shown in Fig. 4a, alcohol exposure produced smaller, rounder, and fragmented mitochondria that had a shorter mean mitochondrial length (1.92 ± 0.86 μm) compared to that of the mitochondria seen in the control group (Fig. 4b). However, the loss of DNA-PKcs preserved mitochondrial network morphology and increased the mitochondrial length to 7.89 ± 1.93 μm. This observation was further supported by western blotting in vitro. As shown in Fig. 4c–g, alcohol increased the expression of mitochondrially located Drp1 (mito-Drp1), which was accompanied by a significant drop in cytoplasmic Drp1. In addition, obvious declines in mitochondrial fusion proteins such as Mfn1 and Opa1 were also noted after chronic alcohol exposure (Fig. 4c–g). To determine whether mitochondrial fission is modulated by DNA-PKcs, FCCP, an activator of mitochondrial fission, was added to DNA-PKcs-depleted cells. As shown in Fig. 4a–g, DNA-PKcs deficiency-mediated fission inhibition was abolished after supplementation with FCCP. This finding suggests that mitochondrial fission is activated by alcohol stress due to DNA-PKcs activation. Similar results were also observed in vivo (Supplementary Fig. S3a–e).

Coimmunofluorescence was used to further analyze the regulatory effect of DNA-PKcs on Drp1-related mitochondrial fission. Mitochondria labeled with Drp1 exhibited larger amounts of free debris in the alcohol-exposed group than in the control group (Fig. 4h). However, in DNA-PKcs-silenced primary hepatocytes, the level of Drp1 in mitochondria clearly decreased, and the mitochondria maintained almost normal morphology with fewer fragments (Fig. 4h). However, the activation of fission via FCCP reintroduced mitochondrial fragments labeled with Drp1 in DNA-PKcs-deleted cells.

To explain the consequences of fission on mitochondrial function, we assessed mtDNA stability and CL oxidation. Mitochondrial genomic stability was reflected by mtDNA copy number and transcription. Alcohol repressed mtDNA copy number and transcription (Supplementary Fig. S4a–c), and this effect was reversed by DNA-PKcs knockdown via the inhibition of mitochondrial fission. In addition, through NAO staining of nonoxidized CL, we found that alcohol-mediated CL oxidation was inhibited by DNA-PKcs deletion; this effect was negated by FCCP treatment (Supplementary Fig. S4d–e). Altogether, the above data confirm that DNA-PKcs deficiency sustains mitochondrial function by suppressing mitochondrial fission.

### Genetic inhibition of DNA-PKcs reverses FUNDC1-related mitophagy

**Fig. 5:**
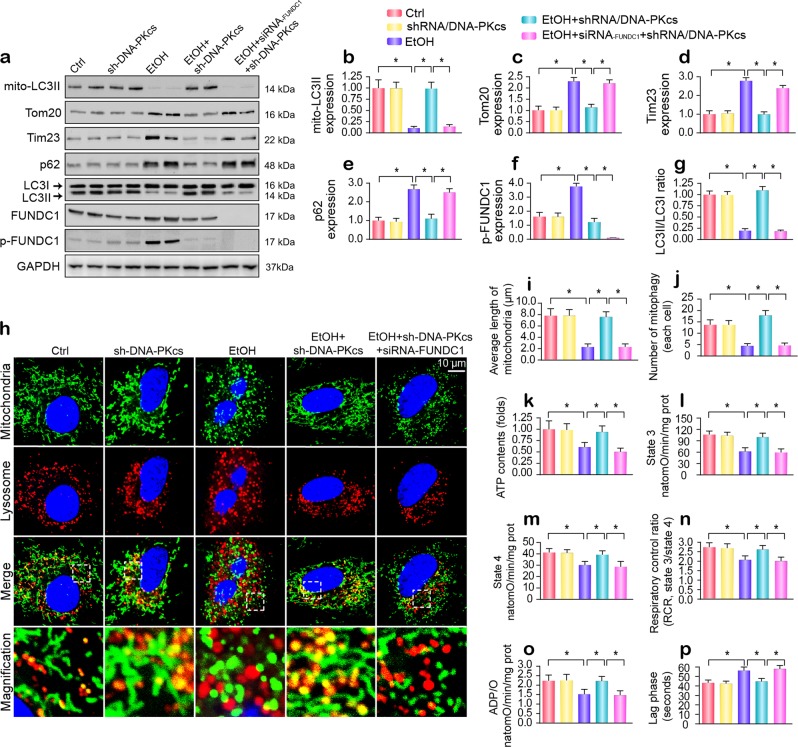
DNA-PKcs negatively regulates mitophagy in hepatocytes. **a**–**g** Immunoblotting assay for mitophagy markers. FUNDC1 siRNA was used to knockdown the expression of FUNDC1. **h**–**j** Costaining of mitochondria and lysosomes. Mitochondria were labeled with Tom-20, and lysosomes were labeled via LAMP1. Organ immunofluorescence was the hallmark of the interaction between mitochondria and lysosomes, which was indicative of mitophagy. FUNDC1 siRNA was used to knockdown the expression of FUNDC1. The mitochondrial length was evaluated to reflect mitochondrial fission. **k** ATP production in primary hepatocytes with DNA-PKcs deletion or FUNDC1 knockdown under alcohol treatment. **l**–**p** Effects of mitophagy on state 3 respiration, state 4 respiration, the respiratory control ratio (RCR [state 3/state 4]), number of nmol of ADP phosphorylated to atoms of oxygen consumed (ADP/O), and the ADP phosphorylation lag phase (time elapsed in the depolarization/repolarization cycle during ADP phosphorylation). The experiments were repeated three times with similar results. The data represent the mean ± standard error of the mean. *p < 0.05.

In response to mitochondrial damage, mitophagy can remove the dysfunctional mitochondria and ensure mitochondrial homeostasis.^6,33^ However, alcohol exposure repressed mitophagy activity, as evidenced by decreased mitochondrial LC3II (mito-LC3II), a reduced LC3II/LC3I ratio, and increased p62 (Fig. 5a–g). In addition, due to mitophagy inhibition, mitochondrial mass, as evidenced by the mitochondrial inner membrane marker Tim-23 and the outer membrane protein Tom-20, was significantly elevated. Nevertheless, the loss of DNA-PKcs reversed mitophagy activity under alcohol stress. Our previous studies showed that FUNDC1 is a novel receptor that is responsible for mitophagy activation via its dephosphorylation at Ser13.^34,35^ However, alcohol obviously enhanced FUNDC1 phosphorylated inactivation (Fig. 5a–g); this change was reversed by DNA-PKcs deletion. Similar results were also observed in vivo (Supplementary Fig. S5a–g). In addition, other mitophagy-related molecules, such as PINK1 and Parkin, were also downregulated by alcohol treatment and reversed to near-normal levels in response to DNA-PKcs deletion (Supplementary Fig. S5a–g). To demonstrate whether FUNDC1 is required for DNA-PKcs-regulated mitophagy, siRNA against FUNDC1 was used in vitro. As shown in Fig. 5a–g, loss of FUNDC1 abolished DNA-PKcs deficiency-mediated mitophagy activation.

The regulatory effects of DNA-PKcs on mitophagy were further examined via coimmunofluorescence using mitochondria and lysosome antibodies. After alcohol treatment, most fragmented mitochondria were unable to be consumed by lysosomes, as demonstrated by the sporadic colocalization of mitochondria and lysosomes (Fig. 5h–j). However, DNA-PKcs deletion contributed to the fusion of mitochondria and lysosomes and thus sustained the mitochondrial network machinery, as shown by the longer length of mitochondria (Fig. 5h–j). Unfortunately, once FUNDC1 was silenced via siRNA transfection, DNA-PKcs deficiency-induced mitophagy was inactivated, a result that was accompanied by an increase in the amount of mitochondrial fragmentation (Fig. 5h–j).

In addition, defective mitophagy was closely associated with an undersupply of ATP (Fig. 5k), which was also accompanied by reductions in the state 3/4 respiratory rate (Fig. 5l, m), ADP phosphorylation (respiratory control ratio) (Fig. 5n), the efficiency of ATP synthesis (ADP/O) (Fig. 5o), and the ADP phosphorylation lag phase (time elapsed in the depolarization/repolarization cycle during ADP phosphorylation) (Fig. 5p), suggesting the protective role played by FUNDC1 mitophagy in sustaining hepatocyte mitochondrial energy metabolism.

### DNA-PKcs regulates Drp1 expression and FUNDC1 phosphorylation through p53

**Fig. 6:**
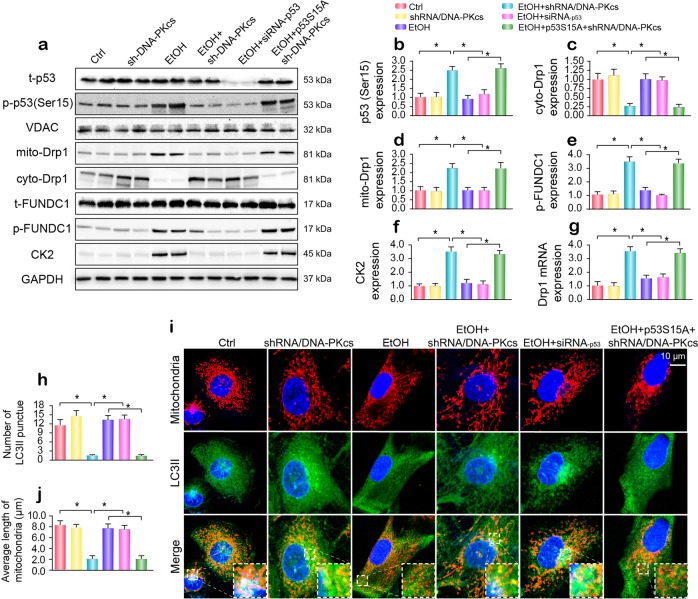
DNA-PKcs-mediated p53 activation promotes Drp1-related mitochondrial fission and suppresses FUNDC1-required mitophagy. **a**–**f** Primary hepatocytes were transfected with p53 siRNA and mutant p53S15A. The Ser15 site of mutant p53S15A was replaced with aspartic acid (permanent phosphorylation of this site). Western blotting was used to analyze Drp1 translocation and FUNDC1 phosphorylation. **g** qPCR was used to evaluate the transcription of Drp1 in response to p53 siRNA and mutant p53S15A. **h**–**j** Immunofluorescence for mitochondria and LC3II. p53 siRNA or mutant p53S15A were transfected into hepatocytes in the presence of ethanol stimulation. The boxed area under each micrograph is enlarged to show the overlap between LC3II puncta and mitochondria. The number of LC3II puncta and the mitochondrial length were measured. The experiments were repeated three times with similar results. The data represent the mean ± standard error of the mean. *p < 0.05.

To explain how DNA-PKcs controls fission and mitophagy, we first focused on p53, which is the primary transcription promoter of Drp1. As shown in Fig. 6a–f, alcohol enhanced the phosphorylation of p53 at Ser15, and this effect was negated by DNA-PKcs deficiency and/or p53 siRNA transfection. To ascertain whether phosphorylated p53 is involved in DNA-PKcs-mediated Drp1-related mitochondrial fission, a p53 gain-of-function assay was carried out via the transfection of a mutant p53 plasmid. The p53 mutant was a constitutively active form of p53 (p53S15A), in which Ser15 was replaced with aspartic acid (permanent phosphorylation of this site). After transfection with p53S15A, phosphorylated p53 was reupregulated (Fig. 6a–f), and this effect was accompanied by increased Drp1 transcription (Fig. 6g) and mitochondrial translocation (Fig. 6a, d). For FUNDC1-related mitophagy, the knockdown of p53 repressed alcohol-mediated p-FUNDC1 upregulation (Fig. 6a–f), which was similar to what was observed after the loss of DNA-PKcs. However, DNA-PKcs deficiency-inhibited FUNDC1 phosphorylation was nullified by p53S15A transfection (Fig. 6a, e). Our previous studies demonstrated that casein kinase 2 (CK2) directly interacts with FUNDC1 and subsequently induces its phosphorylation at Ser13.^19^ In the present study, CK2 was upregulated by alcohol in a p53-dependent manner (Fig. 6a, f). Altogether, the above data indicate that DNA-PKcs regulates Drp1 transcription and FUNDC1 phosphorylation via p53.

To further explain whether p53 is involved in fission and mitophagy, immunofluorescence was performed using Tom-20 and LC3II antibodies. As shown in Fig. 6h–j, alcohol caused mitochondrial debris, which was followed by a drop in LC3II puncta on mitochondria. However, p53 knockdown and/or DNA-PKcs deletion enhanced the colocalization of fragmented mitochondria and LC3II puncta, which was accompanied by an increase in mitochondrial length (Fig. 6h–j). However, p53S15A transfection impaired the communication between mitochondria and lysosomes, leading to the formation of mitochondrial debris in DNA-PKcs-depleted cells.

### The DNA-PKcs/p53 pathway is regulated by NR4A1

**Fig. 7:**
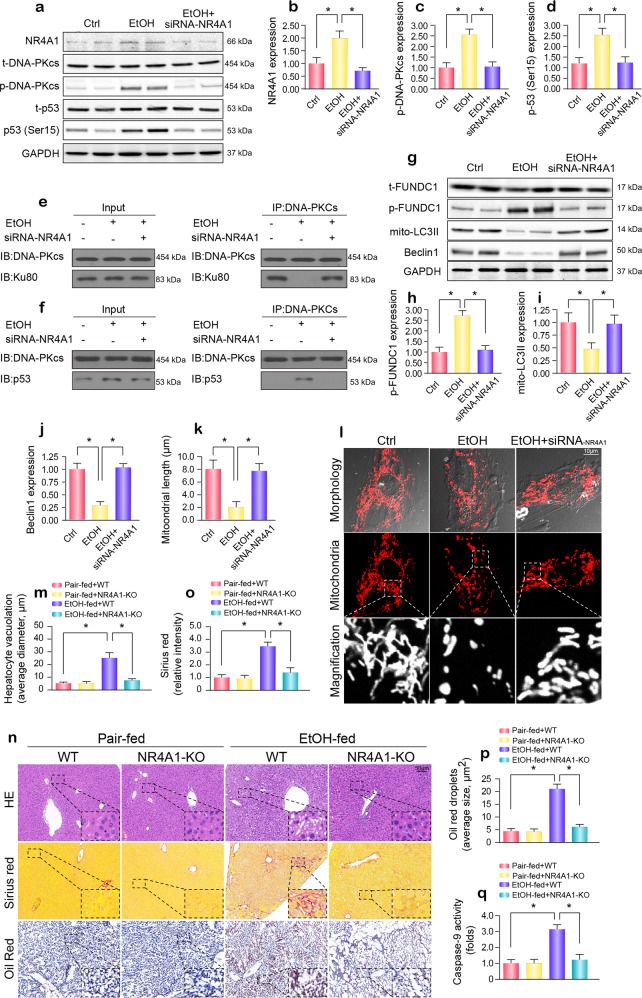
NR4A1 signals DNA-PKcs to interact with p53 and is involved in the development of ARLD. **a**–**d** Proteins were obtained from hepatocytes, and NR4A1 siRNA was used to inhibit NR4A1 expression. Then, the expression of p-DNA-PKcs, p53 and NR4A1 was measured. **e** Co-IP was used to detect the interaction between DNA-PKcs and Ku80. **f** The protein interaction between DNA-PKcs and p53. **g**–**j** The expression of the mitophagy markers phosphorylated FUNDC1, mito-LC3II and Beclin1 in response to NR4A1 knockdown were measured by western blotting. **k**, **l** Mitochondrial fission was observed, and the mitochondrial length was measured. **m**–**p** Paraffin-embedded liver sections were stained with hematoxylin and eosin. Hepatic fibrosis was detected by Sirius Red staining. Frozen liver sections were subjected to Oil Red O staining. **q** Caspase-9 activity was applied to investigate cellular damage. The experiments were repeated three times with similar results. The data represent the mean ± standard error of the mean. *p < 0.05.

Under normal conditions, DNA-PKcs primarily interacts with Ku80 and largely contributes to the recruitment of repair factors to DNA damage. However, in instances of prolonged damage, DNA-PKcs is phosphorylated by orphan nuclear receptor subfamily 4 group A member 1 (NR4A1), thereby leading to the dissociation of DNA-PKcs from Ku80.^23,27^ Subsequently, DNA-PKcs preferentially binds to and activates p53. In our study, NR4A1 expression was increased in response to alcohol treatment (Fig. 7a–d). The loss of NR4A1 suppressed the levels of p-DNA-PKcs and p-p53 (Ser15) (Fig. 7a–d). In addition, by co-IP, we found that DNA-PKcs mainly interacted with Ku80 under normal conditions. However, after exposure to alcohol stress, more p53 was pulled down by DNA-PKcs, whereas little Ku80 interacted with DNA-PKcs. Interestingly, silencing NR4A1 reversed the link between DNA-PKcs and Ku80 and reduced the crosslinking between DNA-PKcs and p53 in the presence of alcohol (Fig. 7e, f). These results demonstrated that the recognition of DNA-PKcs by p53 was NR4A1-dependent. In addition, NR4A1 silencing also reversed mitophagy activity in the presence of alcohol stress, as evidenced by western blotting (Fig. 7g–j). Alcohol-induced mitochondrial fission was also suppressed by NR4A1 silencing, as assessed by immunofluorescence (Fig. 7k, l).

Finally, to elucidate the role of NR4A1 in ARLD, NR4A1 knockout (NR4A1-KO) mice exposed to alcohol intake for 16 weeks were used. Compared to the alcohol-treated WT mice, mice with genetic ablation of NR4A1 exhibited attenuation of hepatocyte vacuolation (Fig. 7m, n), fibrosis (Fig. 7n, o), steatosis (Fig. 7n, p), and caspase-9-related mitochondrial apoptosis (Fig. 7q) induced by alcohol treatment.

## Discussion

**Fig. 8:**
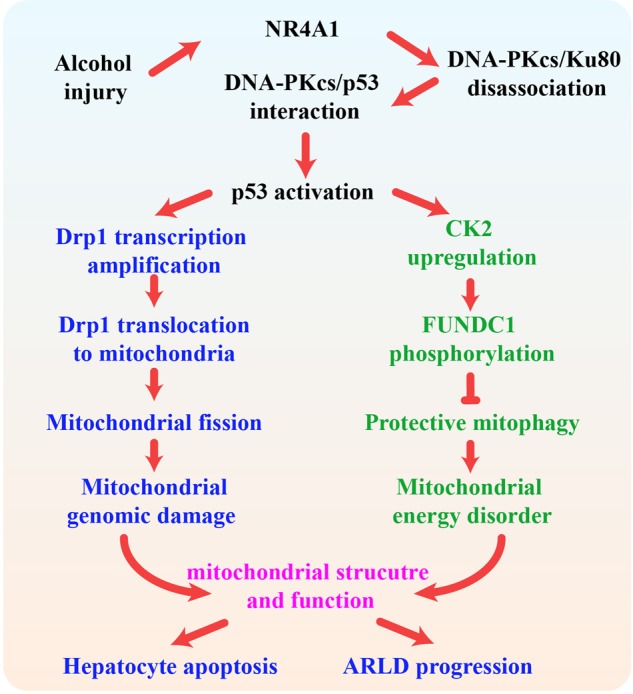
In response to alcohol treatment, NR4A1 was upregulated and blocked the interaction between DNA-PKcs and ku80, promoting the binding of DNA-PKcs to p53. The activated DNA-PKcs/p53 pathway enhanced Drp1 transcription and migration to the mitochondria, inducing mitochondrial fission. Meanwhile, p53 also increased the expression of CK2, impairing FUNDC1-required mitophagy via the phosphorylation of FUNDC1 at Ser13. The excessive fission and badly structured mitophagy were associated with mitochondrial dysfunction, leading to hepatic apoptosis and the progression of ARLD.

Ample evidence has indicated that DNA-PKcs is involved in the development of hepatocellular carcinoma.^36,37^ However, little is known about the role of DNA-PKcs in the progression of ARLD. In the present study, we found that (1) DNA-PKcs was significantly upregulated in the development of ARLD; (2) DNA-PKcs expression positively correlated with liver damage and hepatocyte apoptosis; (3) DNA-PKcs induced hepatic mitochondrial dysfunction by activating mitochondrial fission and repressing mitophagy; (4) p53 was phosphorylated by DNA-PKcs and contributed to Drp1 transcription activation, which led to excessive mitochondrial fission; (5) p53 also upregulated CK2 expression and induced FUNDC1 phosphorylated inactivation at Ser13, which resulted in mitophagy inhibition; (6) NR4A1 was the upstream signal for DNA-PKcs activation and p53 upregulation; (7) NR4A1 blocked the interaction between DNA-PKcs and Ku80 but promoted DNA-PKcs binding to p53; and (8) the genetic ablation of NR4A1 ameliorated the progression and development of ARLD by reversing mitophagy and suppressing fission (Fig. 8). To the best of our knowledge, this is the first study to describe the mechanism underlying the role of DNA-PKcs in hepatocyte death, mitochondrial dysfunction, Drp1-required mitochondrial fission, and FUNDC1-related mitophagy in ARLD.

The key finding in this study was that DNA-PKcs has the ability to regulate mitochondrial homeostasis. A growing collection of data has revealed the deleterious role of mitochondrial damage in the development of ARLD.^38^ Early observations in alcoholic patients included the presence of swollen mitochondria in hepatocytes with mitochondrial DNA breaks, oxidative phosphorylation breakdown, and ROS overproduction.^39,40^ These conclusions correlate well with our results. Furthermore, we illustrated that mitochondrial fission and mitophagy were responsible for mitochondrial dysfunction. DNA-PKcs activated mitochondrial fission and repressed protective mitophagy, thereby leading to mtDNA damage, mitochondrial respiratory inhibition, mROS overproduction, cardiolipin oxidation, membrane potential collapse, pro-apoptotic cyt-c leakage, and mitochondrial apoptosis activation. Although many studies have examined the roles of mitochondrial dynamics and mitophagy in NAFLD, liver transplantation reperfusion injury, and hepatocellular carcinoma,^6,41,42^ the roles of fission and mitophagy in alcohol-induced mitochondrial function and liver injury have been scarcely investigated. Our findings potentially help fill gaps in current knowledge regarding the molecular links between fission, mitophagy, mitochondrial dysfunction, and ARLD.

We determined that p53 is the regulator of fission and mitophagy. DNA-PKcs activated and phosphorylated p53, which enhanced Drp1 transcription, a key factor for mitochondrial fission. In addition, p53 also promoted CK2 expression, which drove FUNDC1-phosphorylated inactivation. Accordingly, we confirmed the regulatory effects of the DNA-PKcs/p53 pathway on Drp1-required fission and FUNDC1-related mitophagy in the setting of ARLD. The impact of fission in inducing cell damage in different liver diseases has been well discussed.^43,44^ However, the role of FUNDC1 mitophagy in ARLD has not been fully investigated. Based on our results, the activation of FUNDC1 mitophagy attenuated alcohol-mediated mitochondrial injury and hepatocyte death. This finding is similar to that of our previous study showing that FUNDC1 mitophagy favors cardiomyocyte survival by attenuating mitochondrial stress in the setting of cardiac ischemia-reperfusion injury.^34,35^ In addition, a recent study demonstrated that the activation of FUNDC1-dependent mitophagy relieves chemical carcinogen diethylnitrosamine-induced hepatocarcinogenesis by interrupting inflammasome activation.^45^ Moreover, FUNDC1 mitophagy also directs skeletal muscle-adipose crosstalk to alleviate dietary obesity.^46^ Therefore, our experiments offer evidence to support the protective role of FUNDC1 in ARLD-induced hepatic damage. These findings may underscore the possibility that FUNDC1 mitophagy acts as an endogenous defender of mitochondrial integrity and hepatocyte homeostasis upon constant alcohol exposure.

DNA-PKcs plays a vital role in maintaining genomic integrity. Under physiological conditions, DNA-PKcs preferentially binds to Ku80^25^ and initiates the DNA-repair system to sustain genome homeostasis and cellular survival.^47,48^ However, after chronic stress or irreversible damage, DNA-PKcs selectively pairs with and strongly activates p53, thereby initiating programmed death signals in mitochondria. Accordingly, the balance between DNA-PKcs/Ku80 and DNA-PKcs/p53 is the primary determinant of cell fate. In the present study, our observations showed that increased NR4A1 is a key factor that drives the DNA-PKcs/p53 axis rather than the DNA-PKcs/Ku80 pathway, suggesting that NR4A1 induces a shift from DNA repair signaling to cellular suicide machinery in the context of ARLD. Although NR4A1 has been linked to the pathogenesis of fatty liver disease^7^ and hepatitis,^49^ this is the first evidence to uncover the pathogenic role exerted by NR4A1 in ARLD, and it may offer a novel insight into treatment for ARLD.

Several limitations exist in our present study. First and perhaps foremost, additional experiments using human samples are necessary to further verify our findings. Second, it remains unknown whether DNA-PKcs modulates other mitophagy receptors in ARLD, such as Bnip3 and Nix. Collectively, the results of our report identified the pathogenic role of DNA-PKcs in mitochondrial dysfunction with respect to ARLD. Mechanistically, DNA-PKcs was regulated by NR4A1 and selectively activated p53, thereby initiating Drp1-required mitochondrial fission and undermining FUNDC1-related mitophagy, which led to hepatocyte death and ARLD development.

## Materials and methods

### Animal model and hepatocyte isolation

The present study was conducted according to the Guide for the Care and Use of Laboratory Animals, which was published by the US National Institutes of Health (NIH Publication No. 85-23, revised 1996) and the guidelines of the University of Wyoming Institutional Animal Use and Care Committee (Laramie, WY). NR4A1 knockout (NR4A1-KO) mice were obtained from the Jackson Laboratory. To specifically knock out DNA-PKcs in the liver, DNA-PKcs^*fl/fl*^ mice were crossed with Alb^*Cre+*^ mice to obtain liver-specific DNA-PKcs knockout (DNA-PKcs^*LKO*^) mice. The generation of DNA-PKcs^*fl/fl*^ mice was reported in our previous study.^7^ These mice (three months old, female) were randomized into ethanol-fed and pair-fed groups and then adapted to a control liquid diet for 16 weeks according to our previous study.^15^ The ethanol-fed groups were allowed free access to an ethanol-containing diet, specifically an isocaloric 4% (vol/vol) alcohol diet with ~24% total calories originating from ethanol. The pair-fed groups were fed a regular liquid diet without alcohol.

Hepatocyte isolation was performed using the Hepatocyte Isolation System (Worthington Biochemical Corporation, Lakewood, NJ, USA) as previously described.^6,7^ In brief, a loose half-square or equivalent knot was tied around the vein. The vena cava was located and opened for drainage just before the portal vein. The perfusion pump containing plain CMF-HBSS was turned on an adjusted to a flow rate of 10–15 mL/min. Tubing was inserted into the portal vein toward the liver. After 7–10 min of CMF-HBSS perfusion, the perfusion solution was switched to the enzyme buffer solution. The liver was perfused with the digestion mixture until it swelled fully, and the liver was fully digested for approximately 20–30 min. At the end of the perfusion, the pump was stopped, the liver was gently placed in a culture dish, and the undigested tissue was removed. Subsequently, viable hepatocytes ﻿were enriched by Percoll gradient centrifugation. In the cell study, primary hepatocytes isolated from WT mice were treated with ethanol for 48 h at a concentration of 100 mM based on our previous research.^15^

### Mitochondrial fission and mitophagy detection

Immunofluorescence was used to observe mitochondrial fission and mitophagy using a mitochondria-specific Tom-20 antibody, a lysosome-specific LAMP1 antibody, and an LC3II antibody under laser scanning confocal microscopy (Nikon A1R, Japan). Western blot analysis of mitophagy-related proteins was also used to quantify mitophagy activity according to our previous studies.^34^ The detail of other materials and methods, including histopathological analysis, immunohistochemistry, immunofluorescence, electron microscopy, qPCR, western blotting, coimmunoprecipitation, cellular apoptosis, oxidative injury, ATP production, mPTP opening, mitochondrial membrane potential (ΔΨm), and mtDNA copy/transcription detection, high-performance thin-layer chromatography analysis of cardiolipin, mitochondrial respiratory function evaluation, mitochondrial calcium ([Ca^2+^]m) detection, and RNA silencing are described in the Supplementary materials.

### Statistical analysis

All data in this study are expressed as the mean ± SEM of at least three independent experiments. Statistical analysis of the differences was performed by one-way analysis of variance (ANOVA) or Student’s *t* test using SPSS 17.0 software. A *P* value < 0.05 was considered statistically significant.

## Supplementary information

Supplementary information
