# Supplementary material for: DNA-PKcs promotes alcohol-related liver disease by activating Drp1-related mitochondrial fission and repressing FUNDC1-required mitophagy
Source: Signal Transduct Target Ther. 2019 Dec 6;4:56. doi: 10.1038/s41392-019-0094-1 (PMC6895206; doi:10.1038/s41392-019-0094-1)
Supplement: Supplementary file 1 — Supplementary information [file 41392_2019_94_MOESM1_ESM.docx]

**Supplementary Information**

(This file contains supplementary methods, supplementary figures S1-S5, and supplementary table S1)

**DNA-PKcs promotes alcohol-related liver disease through activating Drp1-related mitochondrial fission and repressing FUNDC1-required mitophagy**

Hao Zhou^1, 2^, Pingjun Zhu^1^, Jin Wang^1^, ﻿Sam Toan^3^, Jun Ren^2^

^1^Chinese PLA General Hospital, Medical School of Chinese PLA, Beijing 100853, China

^2^Center for Cardiovascular Research and Alternative Medicine, University of Wyoming College of Health Sciences, Laramie, WY 82071, USA

﻿﻿^3^Department of Chemical Engineering, University of Minnesota-Duluth, Duluth, MN 55812, USA

Correspondence to: Dr. Hao Zhou^a,b^ (e-mail: [zhouhao301@outlook.com; zhouhao@plagh.org)](mailto:zhouhao301@outlook.com)), Pingjun Zhu^a^ (e-mail: [zhupingjun@outlook.com](mailto:zhupingjun@outlook.com)), and Jun Ren^b^ (e-mail: [jren@uwyo.edu)](mailto:jren@uwyo.edu))

^a^Chinese PLA General Hospital, Medical School of Chinese PLA, Beijing, China.

^b^Center for Cardiovascular Research and Alternative Medicine, University of Wyoming College of Health Sciences, Laramie, WY 82071 USA

**Running title: DNA-PKcs controls ARLD progression.**

**Supplementary Methods**

**Histopathological analysis, immunohistochemistry and immunoﬂuorescence staining**

Liver tissues were fixed in 4% paraformaldehyde, dehydrated, and embedded in paraffin followed by dehydration in graded ethanol solutions and in toluene. Four-micron-thick sections of the liver were stained with hematoxylin and eosin (H.E.) examined via light microscopy for histopathological analysis. For Oil Red O staining, livers were sliced and snap-frozen in isopentane-cooled liquid nitrogen prior to cutting into 10μm sections with a cryostat. Immunohistochemistry and immunoﬂuorescence staining were conducted on 4-mm sections of liver tissue or cells according to our previous studies^1,2^. In brief, samples were ﬁxed with 4% paraformaldehyde for 10 min, permeabilized with 0.3% Triton X-100 for 5 min, and blocked with 10% goat serum albumin (Invitrogen) for 1 h at room temperature. Specimens were subsequently incubated with primary antibodies overnight, then washed with PBS three times, and incubated with Alexa Fluor 488 donkey anti-rabbit secondary antibody (Invitrogen) for 45 min at room temperature. After being washed, the cells were treated with DAPI for 5 min and analyzed under a ﬂuorescence microscope. The primary antibodies were as follows: MMP9 (1:500, Abcam, #ab119906), VCAM1 (1:500, Abcam, #ab134047), Drp1 (1:500, Cell Signaling Technology, #3455) and cyt-c, (1:500, Abcam, #ab133504). The mitochondrial antibody (Tom20, 1:500, Abcam, #ab186734) and lysosome antibody (Lamp1, 1:500, Abcam, #ab24170) were used to marker the mitochondria and lysosome, respectively.

**ALT and AST measurement.**

Plasma samples were assayed for alanine aminotransferase (ALT) and aspartate aminotransferase (AST) using commercially available enzymatic assay kits (Diagnostic Chemicals) following the manufacturer’s instructions as our previously described^1^.

**Electron microscopy**

The electron microscopy was used to observe the ultrastructure of mitochondria, samples were dehydrated using acetonitrile and graded methanol, embedded in epoxy resin (EMbed-812; Electron Microscopy Sciences, USA) and polymerized at 70°C overnight. Hitachi H600 Electron Microscope (Hitachi, Japan) was used to capture the images.

**Western blotting and co-immunoprecipitation**

After treated, cells were washed with cold PBS and lysed with RIPA buffer containing protease inhibitor cocktail (Thermo Fisher Scientific, Waltham, MA). Lysates were centrifuged at 14,000 g for 15 min at 4˚C. Protein concentration was quantified with Pierce BCA Protein Assay Kit (Thermo Fisher Scientific, Waltham, MA). The 50 μg of the protein extracts were separated by SDS-PAGE and electrotransferred onto nitrocellulose membranes (350 mA for 60 min). Membranes were blocked with 5% fat-free milk in TBS-T buffer for 90 min and incubated overnight at 4˚C with primary antibodies. The primary antibodies for the blots for western blots were as follows: Drp1 (1:1000, Abcam, #ab56788), Beclin1 (1:1000, Cell Signaling Technology, #3738), LC3I/II (1:1000, Cell Signaling Technology, #4108), LC3II (1:1000, Cell Signaling Technology, #3868), total-p53 (1:1000, Cell Signaling Technology, #9282), phospho-p53 (Ser15) (1:1000, Cell Signaling Technology, #9284), total DNA-PKcs (1:1000, Abcam, #ab32566), phospho-DNA-PKcs (Ser2056) (1:1000, Abcam, #ab103970), Tom20 (1:1,000, Abcam, #ab186735), Tim23 (1:1000, Abcam, #ab230253), p62 (1:1000, Cell Signaling Technology, #5114), Bad (1:1,000; Abcam; #ab90435), NR4A1 (1:1000, Cell Signaling Technology, #3960), Bcl2 (1:1000, Cell Signaling Technology, #3498), Bax (1:1000, Cell Signaling Technology, #2772), caspase9 (1:1000, Cell Signaling Technology, #9504), pro-caspase3 (1:1000, Abcam, #ab13847), cleaved caspase3 (1:1000, Abcam, #ab49822), c-IAP (1:1000, Abcam, #ab25939), survivin (1:1000, Cell Signaling Technology, #2808), Fis1 (1:1000, Abcam, #ab71498), Opa1 (1:1000, Abcam, #ab42364), Mfn1 (1:1000, Abcam, #ab57602) CK2 (1:1000, Cell Signaling Technology, #2656), GAPDH (1:1000, Cell Signaling Technology, #5174). The phosphorylated-FUNDC1 (1:500) and total-FUNDC1 (1:1000) polyclonal antibodies were produced by immunizing rabbits with synthesized and purified phosphorylated and nonphosphorylated peptides from FUNDC1 (Abgent, SuZhou, China) according to our previous study^3^. Representative blots were shown from three times experiments and the images were taken with an enhanced chemiluminescence (ECL) reagent.

Co-immunoprecipitation experiments were performed as our previous study described. Brieﬂy, cells were lysed by sonication in PBS with 1% Triton X-100 and incubated with the respective antibodies and protein A/G agarose. The beads were washed using RIPA lysis buffer for at least three times, and then boiled in SDS loading buffer. Immunoprecipitated protein complexes were detected using western blotting.

**qPCR assay**

Total RNA was extracted from the cells using TRIzol ® reagent (Invitrogen Life Technologies, Carlsbad, CA, USA) and was reverse transcribed into a total of 1 µl (60 ng/µl) cDNA using a One‑Step RT‑PCR kit (TransGen Biotech Co., Ltd., Beijing, China), according to our previous study. Quantification of gene expression was performed using an ABI PRISM 7500 Sequence Detection system (Applied Biosystems Life Technologies, Foster City, CA) with SYBR® Green (TransGen Biotech Co., Ltd.). The relative mRNA expression levels were normalized to that of β-actin using the 2^−ΔΔCT^ method. The primer sequences were as follows: IL1 (forward Prime 5′-TTGCTCGAGTGAGTGAGGAT-3′, Reverse Prime 5′-TGTGACAGCGATGGACAGTG-3′), MCP1 (forward Prime 5′-ATGGCAGTGTAGCACAACCA-3′, Reverse Prime 5′-GCGAATGCCATCCCACAGAT-3′), TGFβ (forward Prime 5′-CCTCGTGCTCCCTAGTCTAC-3′, Reverse Prime 5′-AACATCGGAGTGACCTTTGG-3′), TNFα (forward Prime 5′-ACTCAAAGGACTTGGCGGTA-3′, Reverse Prime 5′-AGCCCATTTCTTCCCATTTC-3′), PGC1 (forward Prime 5′-TCGGCGTAAAACGTGTCAAC-3′, Reverse Prime 5′-CCGCCAAGTCCTTTGAGTTT-3′), NFR1 (forward Prime 5′-TGACCCATAGCCATAATATGATTT-3′, Reverse Prime 5′-CTCTACGTTAAACCCTGATACTAA-3′), TFAM (forward Prime 5′--ACACGCCATAATGGCACTCC-3′, Reverse Prime 5′-CAGTCTTGGCAGTGCAGAT-3′), IL8 (forward Prime 5′-TCTCTACCACCTATGGTCGG-3′, Reverse Prime 5′-CACAGGGTTGAGCCAAAAGT-3′), MIP1α (forward Prime 5′-CTCAACATCATGAAGGTCTC-3′, Reverse Prime 5′-GGCATTCAGTTCCAGGTCAG-3′).

**Measurement of markers of oxidative damage**

Mitochondrial ROS, a characterization of oxidant status, are involved in the injury of hepatocyte in the setting of ALD. The mROS measurement was conducted using a MitoSOX red mitochondrial superoxide indicator (Molecular Probes, USA). Malondialdehyde (MDA), an end product of peroxidation of cell membrane lipids caused by oxidative free radicals, is considered a reliable marker of oxidative damage. Glutathione (GSH) and superoxide dismutase (SOD) play important roles in clearing ROS, thereby protecting cells from oxidative damage. The MDA content, SOD activity, and GSH concentration were measured using commercial kits (Sigma, USA) following the manufacturer's instructions.

**Caspase3/9 activity and TUNEL assay**

Caspase3 and 9 activity were measured via a commercial kit (Beyotime, China) as our previous study described. TUNEL assay was used to detect the apoptosis cell according to the manufacturer’s protocol. The degree of apoptosis was calculated as the number of TUNEL-positive cells per 500 cells nuclei.

**Mitochondrial membrane potential (ΔΨm)**

The mitochondrial transmembrane potential was analyzed using a mitochondrial membrane potential detection kit (JC-1) (Beyotime Institute of Biotechnology) according to the manufacturer's instructions. Brieﬂy, cells were washed with ice-cold PBS and then stained with 2.5 g/ml JC-1 for 30 min at 37°C. After being washed with binding buffer, the cells were analyzed by ﬂuorescence microscopy. Results are presented as relative aggregate-to-monomer (red/green) ﬂuorescence intensity ratio.

**ATP production, mPTP opening assays and NAO staining**

To investigate the function of mitochondria, ATP production was examined by the luciferinluciferase method^40^ following the protocol of ATP detection kit (Beyotime, China). The opening of the mPTP was visualized as a rapid dissipation of tetramethylrhodamine ethyl ester ﬂuorescence. Arbitrary mPTP opening time was determined as the time when tetramethylrhodamine ethyl ester ﬂuorescence intensity decreased by half between initial and residual ﬂuorescence intensity according to our previous study. Staining with 10-N-nonyl acridine orange (NAO; 2 mmol/L, Molecular Probes) was used to observe the cardiolipin oxidation. Images were captured using a ﬂuorescence microscope (OLYMPUS DX51; Olympus, Tokyo, Japan) and were analyzed with Image-Pro Plus 6.0 (Media Cybernetics, Rockville, MD) to obtain the mean densities of the region of interest, which was normalized to that of the control group.

**The mtDNA copy numbers and transcription level detection**

The relative amounts of mitochondrial DNA (mtDNA) and nuclear DNA (nDNA) content were used to assess the mtDNA copy numbers via reverse transcription polymerase chain reaction based on our previous study. The mtDNA and nuclear amplicons were generated from a complex IV segment and GAPDH segment, respectively. The mtDNA primers were 5′-CAGGATTCTTCTGAGCGTTCTATCA-3′ and 5′-AATTCCTGTTGGAGGTCAGCA-3′. The GAPDH primers, chosen as the internal standards, were 5′-ACGGCAAATTCAACGGCACAGTCA-3′ and 5′-ACGGCAAATTCAACGGCACAGTCA-3′.

The transcript level of mtDNA was reﬂected by two different components: NADH dehydrogenase subunit 1 (ND1) and cytochrome c oxidase subunit I (COX-I). The primers for COX-I were 5′-GAAGAGACAGTGTTTCATGTGGTGT-3′ and 5′-TCCTGGGCCTTTCAGGAATA-3′. The primers for ND1 were 5′-ATGGTCAGTCTGTCATGGTGGAAC-3′ and 5′-GCATAGCACAAGCAGCGACAAC-3′. GAPDH was selected as the internal standard. The experiments were repeated 3 times with triplicates of each sample.

**Mitochondrial respiratory assays**

Mitochondrial respiration was initiated by adding glutamate/malate to a ﬁnal concentration of 5 and 2.5 mmol/L, respectively. State 3 respiration was initiated by adding ADP (150 nmol/L); state 4 was measured as the rate of oxygen consumption after ADP phosphorylation. The respiratory control ratio (state 3/state 4) and the ADP/O ratio (number of nmol ADP phosphorylated to atoms of oxygen consumed) were calculated as previously described.

**Detection of mitochondrial calcium ([Ca^2+^]m)**

For analysis of [Ca^2+^]m, Rhod-2 (Molecular Probes) was used and the images were captured by confocal microscopy per our previous study. Fluorescence intensity of Rhod-2 was measured by excitation wavelengths of 550 nm and emission wavelengths of 570 nm, respectively. Data (F/F0) were obtained by dividing fluorescence intensity (F) by (F0) at resting level (t = 0) which was normalized by control groups.

**Cardiolipin extraction and high-performance thin-layer chromatography analysis**

The mitochondrial was used to obtain cardiolipin by and high-performance thin-layer chromatography with an electrospray ionization source and a linear ion trap mass spectrometer (LXQ Thermo-Fisher). To avoid the oxidation of phospholipids during separation, chloroform/methanol (2/1, v/v) containing 0.05 % BHT as antioxidant was added. The cardiolipin and its oxidized molecular species were extracted based on our report and separated on a normal phase column (Luna 3 μm Silica 100A, 150 × 2mm, Phenomenex, Torrance CA) with a flow rate of 0.2 mL/min applying a gradient elution using solvents containing 5 mM CH_3_COONH_4_ (A-n-hexane : 2-propanol : water, 43:57:1 (v/v/v); and B-n-hexane : 2-propanol : water, 43:57:10 (v/v/v). Analysis of (hydroperoxyand hydroxy-) oxidized phospholipid species was performed as our previous study described.

**RNAi assay**

The shRNA specific against the expression of DNA-PKcs or control shRNA were transfected into hepatocyte to inhibit the expression of DNA-PKcs. To suppress the p53, NR4A1 and FUNDC1 expression, siRNA was used according to our previous study. The siRNAs targeting p53, NR4A1 and FUNDC1 were purchased from Santa Cruz Biotechnology.

**Statistical analysis**

All data in this study are expressed as the mean ± SEM of at least three independent experiments. Statistical analysis of differences was performed by one-way analysis of variance (ANOVA) or student t test using SPSS 17.0 software. P value less than 0.05 was considered statistically significant.

**Supplementary Figures**

**
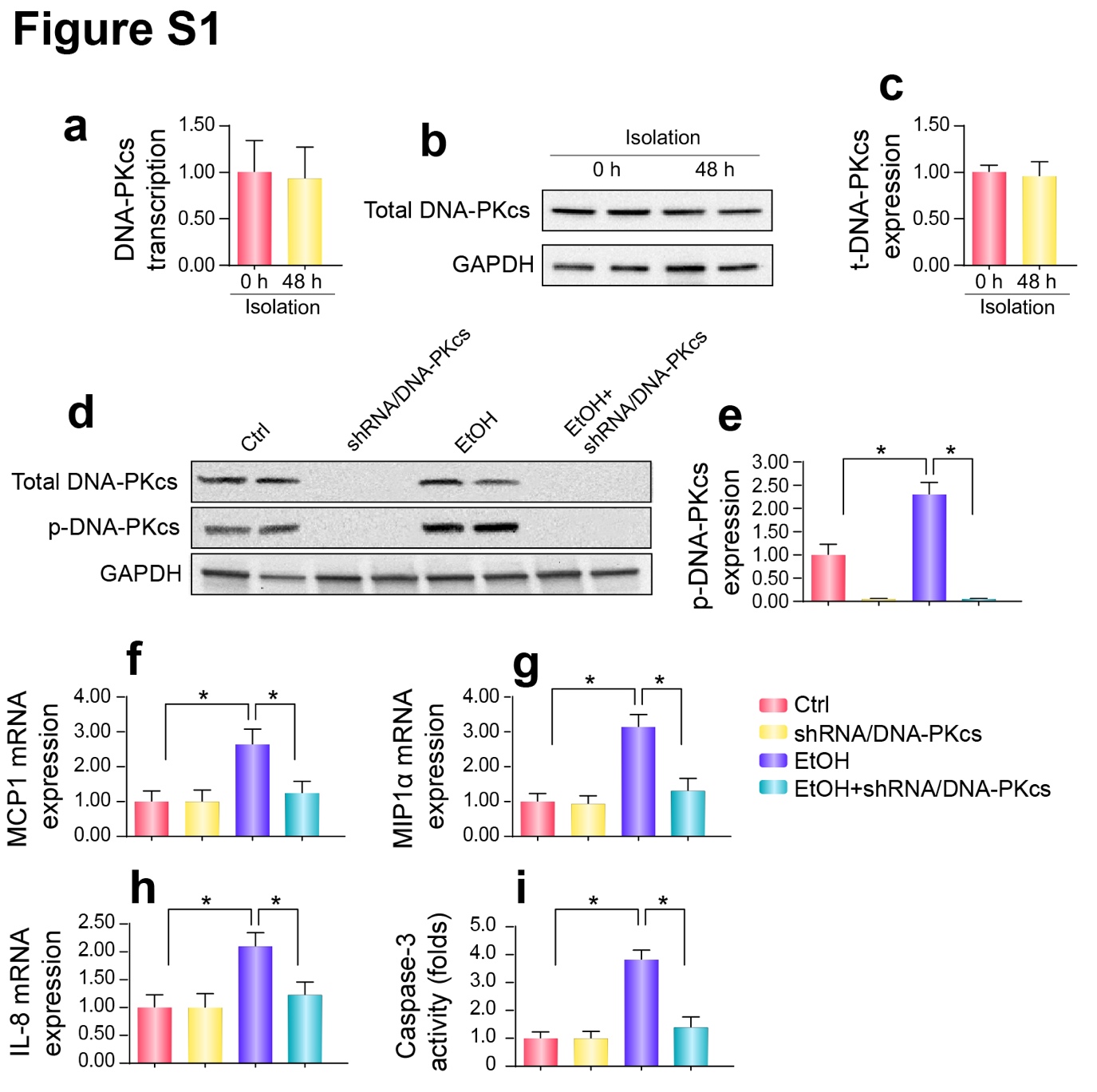
**

**FigureS1. DNA-PKcs-dependent pathway is required for the development of chronic ethanol-induced liver disease.**

**a-c** Primary hepatocytes were isolated from WT mice. After 48 hours isolation, the transcription and expression of DNA-PKcs were determined via qPCR and western blot, respectively.

**d-e** Primary hepatocytes isolated from WT mice were transfected with shRNA/DNA-PKcs. Then, these cells were maintained in culture media with ethanol treatment for 48 hours at 100 mM. Western blots were used to detect expression of DNA-PKcs.

**f-h** The qPCR assay was used to explore the changes of MCP1, MIP1α and IL-8 transcription.

**i** The caspase-3 activity was used to detect hepatic apoptosis under alcohol treatment.

Experiments were repeated three times with similar results. The data represent the mean±standard error of the mean. **p*< 0.05.

**
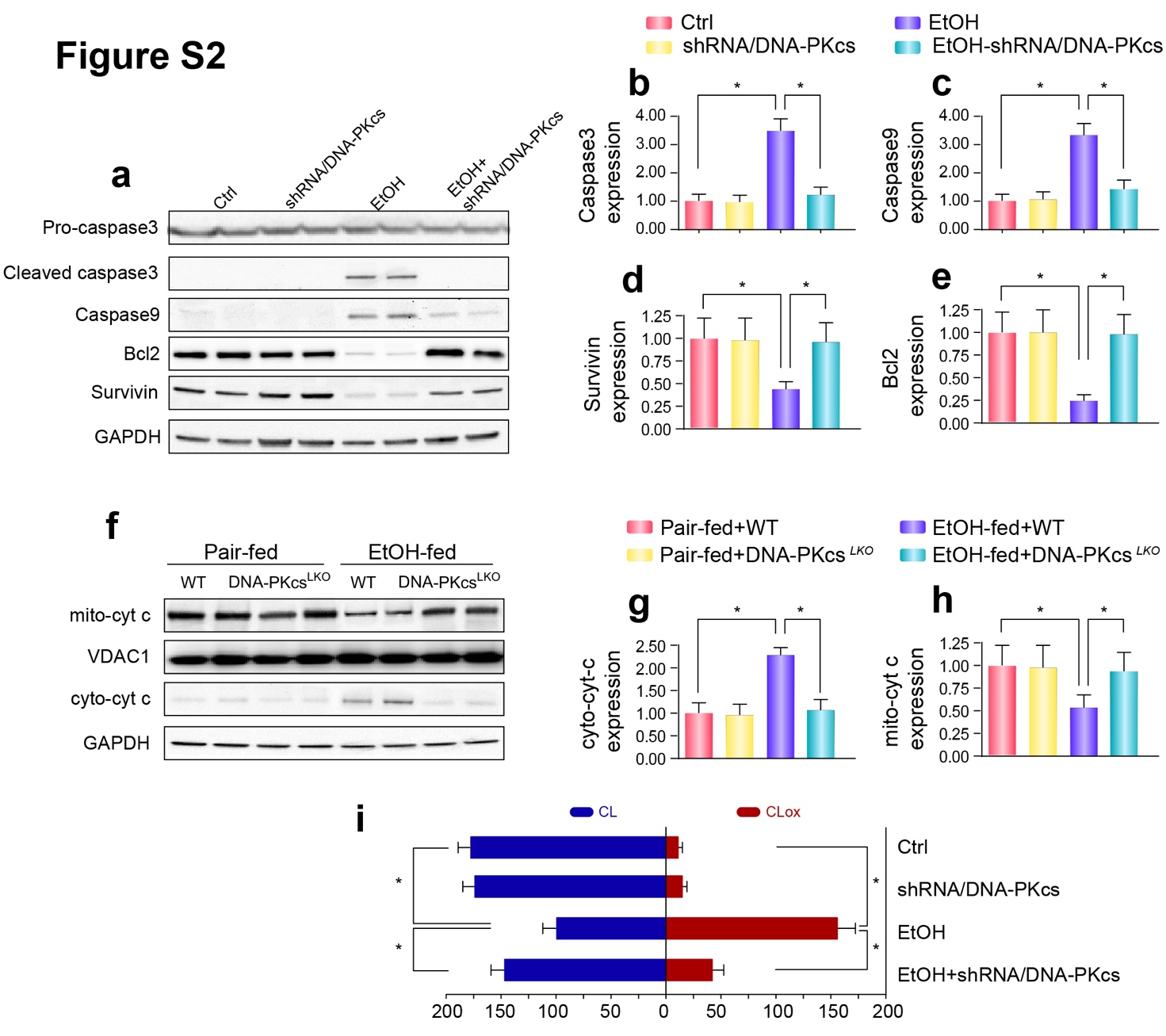
**

**FigureS2 Alcohol treatment induces the cellular apoptosis in hepatocytes.**

**a-e** Western blot analysis of cell lysates from hepatocytes transfected with shRNA/DNA-PKcs in the presence of ethanol stimulation.

**f-h** Tissue lysates from WT and DNA-PKcs*^LKO^* livers untreated or treated with alcohol were analyzed using western blots to determine the cellular location of cyt-c.

**i** The evaluation of number of non-oxidized and oxidized molecular species of CL in hepatocytes. Experiments were repeated three times with similar results.

The data represent the mean±standard error of the mean. **p*< 0.05.

**
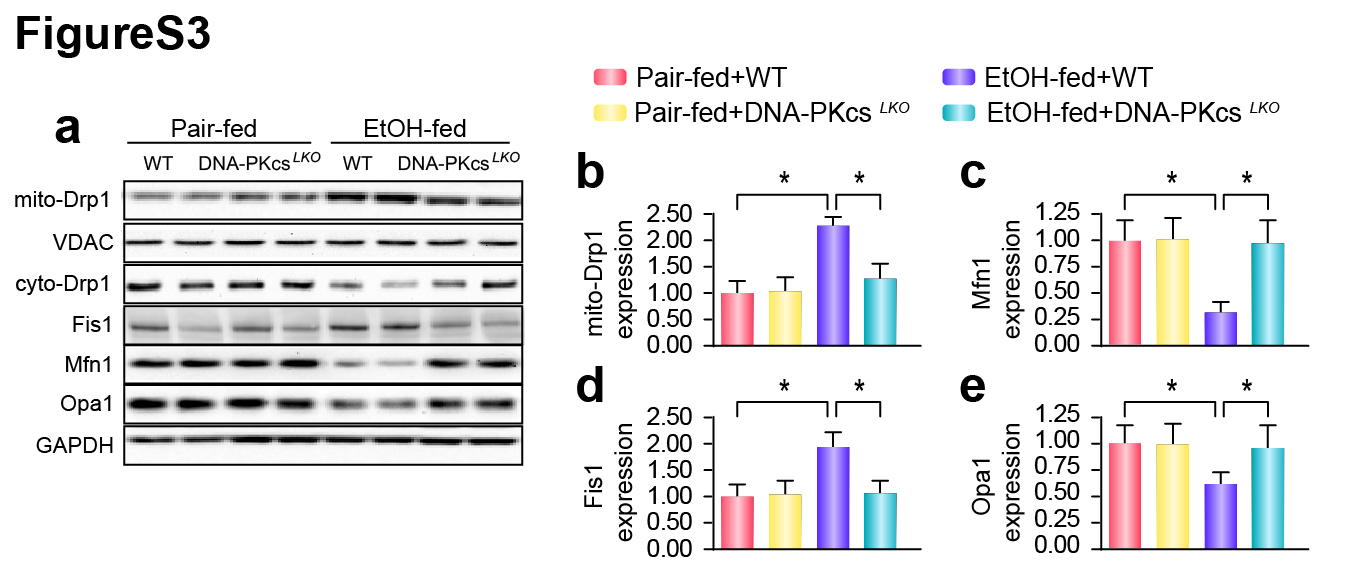
**

**FigureS3 Alcohol regulates the mitochondrial fission.**

**a-e** Western blots were used to examine tissue proteins related to mitochondrial fission and fusion *in vivo*.

Experiments were repeated three times with similar results. The data represent the mean±standard error of the mean. **p*< 0.05.


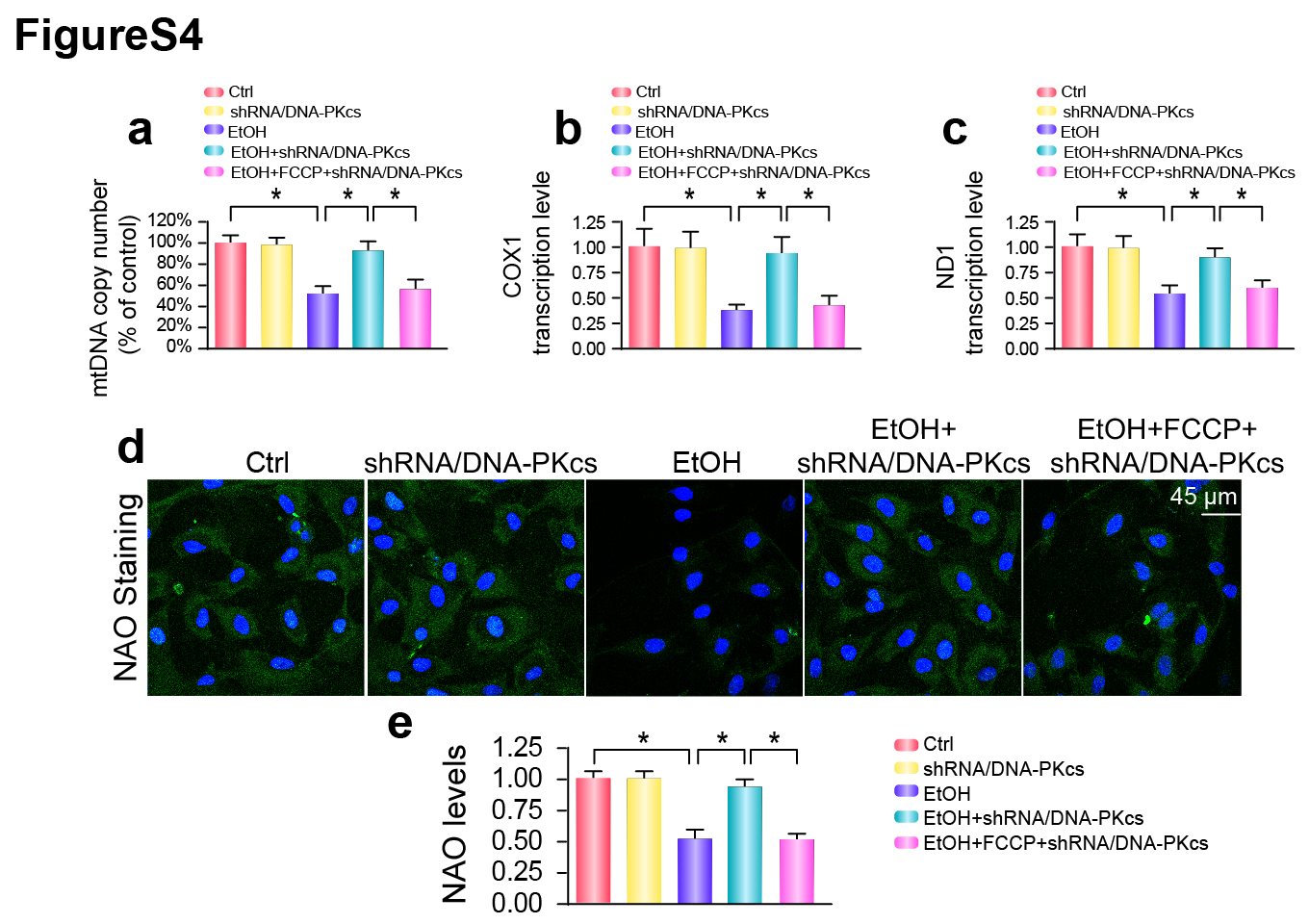


**FigureS4** **Mitochondrial fission promotes mitochondrial dysfunction.**

**a** The mtDNA copy number was assessed by a complex IV segment.

**b-c** The transcript level of mtDNA was reﬂected by two different components: NADH dehydrogenase subunit 1 (ND1) and cytochrome *c* oxidase subunit I (COX I).

**d-e** The changes in 10-N-nonyl acridine orange (NAO) ﬂuorescence indicated cardiolipin (CL) oxidation. In normal cells, NAO interacted with non-oxidized cardiolipin and produced a characteristic green fluorescence. However, after cardiolipin was oxidized, NAO could not bind to it.

Experiments were repeated three times with similar results. The data represent the mean±standard error of the mean. **p*< 0.05.


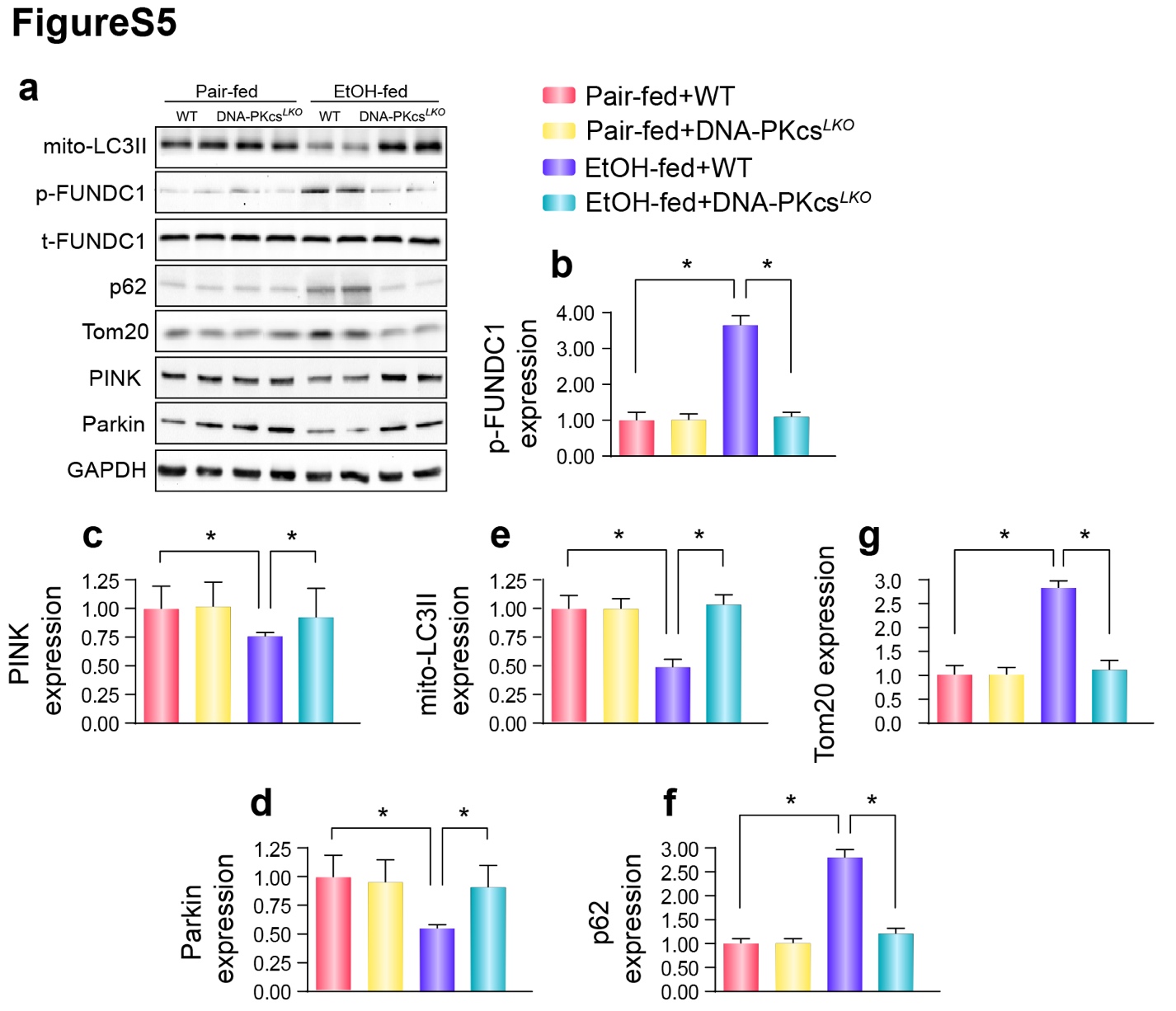


**Figure S5 Alcohol regulates FUNDC1-related mitophagy via DNA-PKcs.**

**a-g** Western blots were used to examine tissue proteins related to mitophagy *in vivo*. Experiments were repeated three times with similar results.

The data represent the mean±standard error of the mean. **p*< 0.05.

|  | WT | DNA-PKcs*^LKO^* | WT+EtOH | DNA-PKcs*^LKO^*+EtOH | |
| --- | --- | --- | --- | --- | --- |
| Body Weight (g) | 28.7±3.3 | 28.9±3.1 | 32.2±6.9 | | 29.3±3.6 |
| Liver Weight (g) | 1.59±0.36 | 1.59±0.31 | 1.96±0.43* | | 1.62±0.29# |
| Liver/body Weight (mg/g) | 55.4±8.2 | 55.0±6.7 | 60.9±8.9* | | 55.2±5.8# |
| Heart weight (mg) | 151±26 | 148±21 | 188±33* | | 154±18# |
| Heart/body weight (mg/g) | 5.26±0.98 | 5.12±1.05 | 5.83±1.23* | | 5.25±0.78# |
| Kidney weight (g) | 0.39±0.05 | 0.39±0.06 | 0.41±0.08 | | 0.39±0.07 |
| Kidney/body weight (mg/g) | 13.6±1.5 | 13.5±1.3 | 12.7±1.6 | | 13.3±1.8 |
| Serum ALT (U/L) | 64±10 | 63±9 | 79±8* | | 66±7# |
| Serum AST (U/L) | 109±12 | 108±11 | 164±21* | | 117±16# |
| AST/ALT ratio | 1.70±0.16 | 1.71±0.13 | 2.08±0.18* | | 1.77±0.14# |

**Supplementary Table S1: Biometric properties in WT and DNA-PKcs*^LKO^* mice with or without an alcohol diet (4%) for about 16 weeks.**

Mean ± SD, n = 6 mice per group. **p*＜0.05 vs. WT group, #*p*＜0.05 vs. EtOH group

**References:**

1 Guo, R., Xu, X., Babcock, S. A., Zhang, Y. & Ren, J. Aldehyde dedydrogenase-2 plays a beneficial role in ameliorating chronic alcohol-induced hepatic steatosis and inflammation through regulation of autophagy. *J. Hepatol.* **62**, 647-656 (2015).

2 Xu, X., Hueckstaedt, L. K. & Ren, J. Deficiency of insulin-like growth factor 1 attenuates aging-induced changes in hepatic function: role of autophagy. *J. Hepatol.* **59**, 308-317 (2013).

3 Zhou, H. *et al.* Ripk3 induces mitochondrial apoptosis via inhibition of FUNDC1 mitophagy in cardiac IR injury. *Redox Biol.* **13**, 498-507 (2017).
